# The expression of HER2/neu in patients with lung cancer and its associated factors

**DOI:** 10.1111/crj.13572

**Published:** 2023-01-08

**Authors:** Mohammadreza Lashkarizadeh, Mahdiyeh Lashkarizadeh, Meead Nikian, Maryam Kouhestani Parizi

**Affiliations:** ^1^ Department of General Surgery, School of Medicine Kerman University of Medical Sciences Kerman Iran; ^2^ Pathology and Stem Cell Research Center, Department of Pathology, School of Medicine Kerman University of Medical Sciences Kerman Iran

**Keywords:** erbB‐2, genes, lung neoplasms, prognosis

## Abstract

**Background and objectives:**

Lung cancer is the most common cause of cancer‐related death worldwide in both sexes. Evidence suggests the role of genetic factors in lung cancer. Studying of such factors can help understand the cancer prognosis. Overexpression of the human epidermal growth factor receptor‐2 (HER2/neu) protein is considered an important prognostic factor in breast cancer, but its role has not been confirmed in lung cancer. Therefore, the present study aimed to investigate the role of its expression in patients with lung cancer.

**Materials and methods:**

In this cross‐sectional study, patients aged >18 years who were referred to Afzalipoor Hospital, Kerman, Iran, from 2016 to 2017, and were diagnosed with lung cancer were enrolled into the study if they had a pathological sample of their cancerous lung. Their demographics were recorded, and the sample was sectioned and stained to measure HER2/neu gene expression according to DAKO instructions using heat‐induced antigen retrieval (HIER) enzyme marker.

**Results:**

The samples of 100 patients with lung cancer were evaluated (84% men and 16% women) with a mean age of 61.34 years (standard deviation of 12.51 years). HER2/neu expression was significantly associated with the type of cancer and was highest in adenocarcinoma and zero in small cell carcinoma (*P* < 0.001), but not with patients' sex, age, smoking status and family history of cancer (*P* > 0.05).

**Conclusion:**

These results emphasized the overexpression of HER2/neu in different types of lung cancer, which can be used further for therapeutic purposes. The results showed that HER2/neu was overexpressed not only in adenocarcinoma but also in other types, like squamous cell carcinoma. Therefore, all subtypes of non‐small cell lung carcinoma should be considered for anti‐HER2 therapies, and further research is required for small cell lung carcinoma.

## Graphical abstract

In this report, we present data from evaluation of HER2/neu overexpression in 100 patients who diagnosed with lung cancer. The results showed that HER2/neu was overexpressed not only in adenocarcinoma but also in other types, like squamous cell carcinoma.

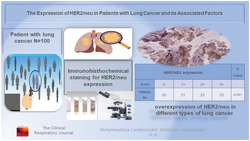

## 1 INTRODUCTION

Lung cancer has been the most common cause of cancer‐related death worldwide for the past several years,^1^ and despite a substantial increase in the incidence of lung cancer in the past four decades, the medical advancement has only resulted in less than 21% increase in the patients' survival rate of this cancer.^2^ The low 5‐year survival rate of lung cancer (about 15.6%) is mainly because most of the cases are diagnosed at advanced stages with metastasis to several organs, although cases diagnosed at early stages have a much better survival rate.^3^ Tumor's growth and metastasis also depend on tumor type, such as small cell lung cancer (SCLC) with a rapid growth rate, lymph node and remote metastasis, and non‐small cell lung carcinoma (NSCLC), which constitutes about 85% of all cancer types and includes adenocarcinoma, squamous cell carcinoma (SCC) and large cell carcinoma, according to which the treatment modality and prognosis differ.^4^

In addition to TNM staging (tumor size, lymph node and remote metastasis), considered the most important prognostic factor in cancers,^5^ determined by radiological imaging techniques, molecular studies have revealed the role of overexpression and overactivation of several genetic factors in the prognosis of lung cancer. They include mutations of epidermal growth factor receptor (*EGFR*), Kirsten rat sarcoma (*KRAS*) and alterations of anaplastic lymphoma kinase (*ALK*).^6^, ^7^ Human epidermal growth factor receptor‐2 (HER2; also known as ERBB2 or HER2/neu) can lead to cell proliferation and tumorigenesis through their tyrosine kinase activity.^8^ With the advent of tyrosine kinase inhibitors (TKIs), anti‐HER2 therapy has been suggested to tackle this disease. Accordingly, it has been suggested that genetic profiling of patients can help identify the most effective treatment modality and thus improve patients' survival.^9^, ^10^

Overexpression of HER2/neu has been found in 10%–30% of different types of cancers, like breast cancers and gastric/gastroesophageal cancers, with prognostic and predictive value.^8^ Evidence suggests that lung cancer is more common in women and Asians.^11^ However, studies addressing HER2/neu expression in patients with lung cancer have only included patients with one specific type of cancer, such as adenocarcinoma,^12^ and other types of NSCLC,^13^, ^14^ mainly with small sample size; and the prognostic value of HER2/neu overexpression in different lung cancer types has to be yet determined.^15^, ^16^ As some suggest that HER2/neu overexpression is observed more commonly in Asians,^11^ more studies are required, especially on this population. Therefore, the present study aimed to investigate the role of HER2/neu expression in a sample of adult patients with different types of lung cancer.

## 2 MATERIALS AND METHODS

### 2.1 Study design

The protocol of the present cross‐sectional study was approved by the Ethics Committee of Kerman University of Medical Sciences (code: IR.KMU.RCE.1395.137). The ethical considerations of the latest version of Helsinki's Declaration on human studies were met throughout the study phases. Adult patients (aged >18 years) who referred to Afzalipoor Hospital, Kerman, Iran, from 2016 until 2017, were diagnosed with lung cancer by the pathological report and had a fixed pathological sample at the hospital's records were selected for enrolment into the study, if they had complete medical records (*N* = 100). The patients' demographics, including age, sex and family history of lung cancer, as well as smoking status and the type of cancer, were recorded from the medical records of the hospital, and the samples were sent to the laboratory for measurement of HER2/neu gene expression.

**FIGURE 1:**
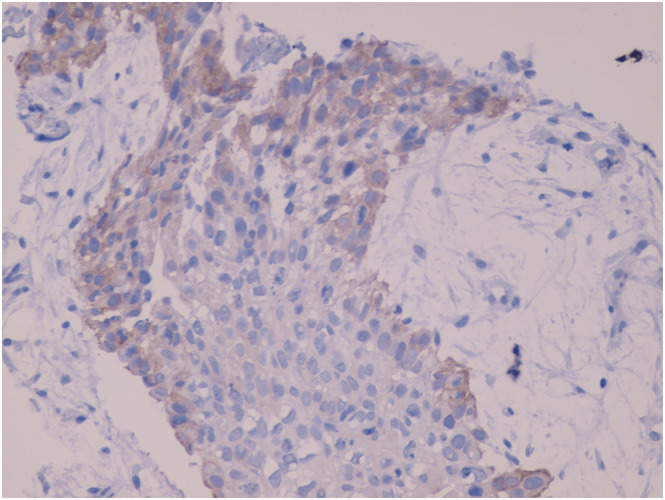
HER2 cells showing immunostaining score 1^+^, low and incomplete membrane marking in less than 10% of the tumoural cells (40×)

**FIGURE 2:**
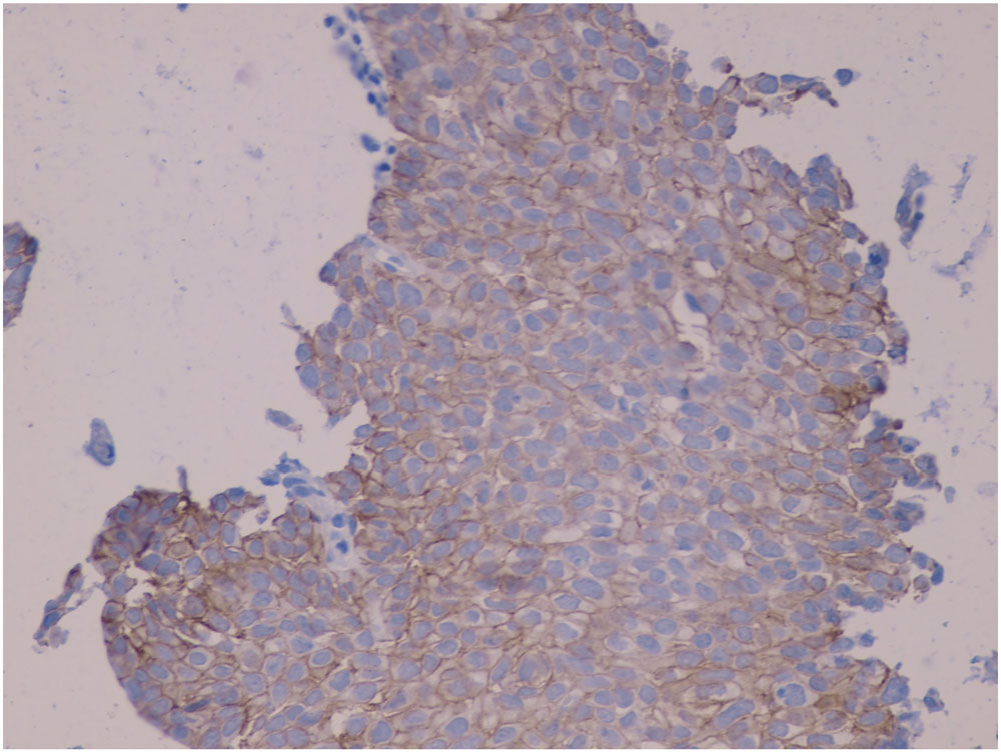
HER2 cells showing immunostaining score 2^+^ incomplete, circumferential membrane staining within >10% of tumor cells (40×)

**FIGURE 3:**
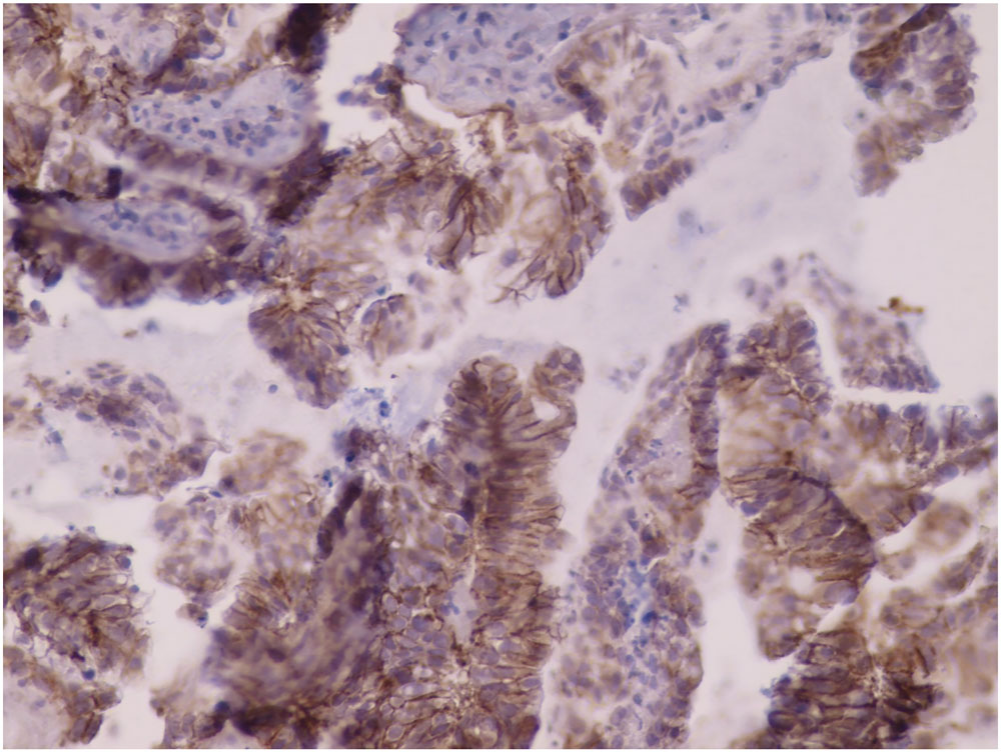
HER2 cells showing immunostaining score 3^+^ complete and intense circumferential membrane staining within >10% of tumor cells (40×)

Paraffin blocks were sectioned into 4‐μm sections. All slides were deparaffinized and hydrated by xylene and graded series of ethanol, respectively. For antigen retrieval of tissue, the slides were immersed in a microwaved retrieval solution for 20 min. For immunohistochemical (IHC) staining, all slides were exposed with mouse monoclonal HER2/neu antibody, IgG1 isotype, diluted at 1:300–1:600 (DAKO, Denmark) and incubated for 30 min at room temperature. The slides were treated with diluted peroxidase‐conjugated secondary antibody and incubated for 30 min at room temperature in a humidified chamber. In the final step, for detection of immune reaction, diaminobenzidine solution was added to all slides and counterstained with haematoxylin. HER2/neu expression positivity was determined based on the American Society of Clinical Oncology/College of American Pathologists breast cancer guidelines as follows: The results of IHC with complete and intense circumferential membrane staining within >10% of tumor cells are considered 3^+^ and strongly positive for HER2 and considered 2^+^ if there is incomplete and/or weak or moderate circumferential membrane staining within >10% of tumor cells and reported as equivocal. Faint or barely perceptible, incomplete membrane staining within >10% of tumor cells are considered 1^+^ and reported as negative. IHC 0 refers to no staining or faint or barely perceptible, incomplete membrane staining within <10% of tumor cells and reported as negative. A sample of IHC 1, 2 and 3 is shown in Figures 1, 2, 3.

### 2.2 Statistical analysis

The results were described by frequency (percentage) and mean ± standard deviation (SD). The variables were compared based on HER2/neu positivity; the qualitative variables were tested using chi‐square test, and the quantitative variables were tested using one‐way ANOVA. For the statistical analysis, the statistical software IBM SPSS Statistics for Windows, Version 21.0 (IBM Corp. 2012. Armonk, NY: IBM Corp.) was used. *P* values of 0.05 or less were considered statistically significant.

## 3 RESULTS

The samples of 100 patients with lung cancer were evaluated (84% men and 16% women). HER2/neu expression was strongly positive in 23% of the patients and equivocal in 18% of the patients; the frequency of patients' sex distribution was not different according to the different degrees of HER2/neu expression (*P* = 0.424; Table 1). The mean ± SD of the patients' age was 61.34 ± 12.51 years (minimum of 34 and maximum of 93 years), and there was no difference in mean age between the patients with different degrees of HER2/neu expression (*P* = 0.215; Table 1).

**TABLE 1:** The frequency of HER2/neu expression based on the patients' characteristics

		HER2/NEU expression				*P* value
		0	1+	2+	3+	
Patients' sex, no. or %	Male	29	16	18	21	0.424^a^
	Female	4	5	5	2	
Patients' age (years), mean ± SD		58.96 ± 13.50	65.85 ± 10.20	62.30 ± 12.94	59.65 ± 12.03	0.215
Smoking status, no. or %	Smoker	28	17	21	18	0.65^a^
	Non‐smoker	5	4	2	5	
Family history, no. or %	Positive	4	0	0	1	0.119^a^
	Negative	29	21	23	22	
Cancer type, no. or %	Small cell carcinoma	15	0	0	0	<0.001^a^
	Adenocarcinoma	4	13	14	16	
	Squamous cell carcinoma	14	8	9	7	
Total		33	21	23	23	

^a^ The results of chi‐square test. All tests were considered significant at *P* < 0.05.

The frequency of cancer types and their categories based on the frequency of HER2/neu expression are shown in Table 1. As indicated, the frequency of HER2/neu expression was significantly different based on the type of cancer, and higher frequencies of 3^+^ and 2^+^ HER2 were observed in adenocarcinoma, whereas all 15 patients with small cell carcinoma had IHC 0 (*P* < 0.001).

## 4 DISCUSSION

In the present study, HER2/neu expression was examined in 100 patients with different types of lung cancers; SCLC (15%), adenocarcinoma (47%), and SCC (38%). The results showed that HER2/neu expression was significantly different based on the type of cancer and was highest in adenocarcinoma and zero in small cell carcinoma. This difference in the HER2/neu overexpression based on tumor type was an important finding of this study, as a review of studies showed that the previous studies have only focused on patients with one type of cancer and have especially focused on adenocarcinoma.^15^ Studying 1478 patients with adenocarcinoma using IHC staining showed HER2/neu overexpression in 6% (25 cases),^17^ which is much lower than that of the adenocarcinoma group in our study. Li et al have also reported HER2/neu overexpression in 3.5% of 224 patients with adenocarcinoma,^11^ which is then again fewer than that of the present study. This difference could be because of the differences in patient and disease characteristics of the study populations, such as age and ethnicity/race, as well as the accuracy of the assessment method used for determination of HER2/neu overexpression. In another sample of 3800 patients with lung cancer, HER2/neu was positive in only 1.7% of patients (*N* = 65), all of which had adenocarcinoma and were mainly women, non‐smoker and 50% at Stage IV.^12^ These results are consistent with that of ours as we found no case of HER2/neu expression in patients with SCLC. However, although about half of the cases with HER2/neu expression scores 1, 2 and 3 were observed in patients with adenocarcinoma, which is inconsistent with the results of the study by Mazieres et al.^12^ In addition to the assessment method used for HER2/neu overexpression, another source of difference among studies is the cut‐off used for reporting HER2/neu expression as positive. We have reported the scores, whereas some others have only reported HER2/neu overexpression.^11^, ^12^, ^17^ Similar to the classification of HER2/neu expression scores in our study, Takenaka et al evaluated HER2/neu overexpression by IHC staining in 159 adenocarcinomas and 77 SCCs, and the results showed a score of 0, 1, 2 and 3 in 76.1%, 10%, 10.7% and 3.1% of patients with adenocarcinoma and in 96.1%, 1.2%, 1.2% and 1.2% of patients with SCC, respectively.^13^ The frequencies reported in their study differ from that of the present study, especially considering SCC, as they have only observed scores 1, 2 and 3 only in one patient,^13^ while we observed them in eight, nine and seven patients with SCC, respectively, although the number of patients with SCC in their study was fewer than that of ours. Nevertheless, their results confirm the results of our study considering different rates of HER2/neu expression positivity in patients with adenocarcinoma and SCC. Considering SCLC, the results of our study showed no case of HER2/neu overexpression at any scores in patients with SCLC. This is while previous studies have reported HER2/neu overexpression in patients with SCLC^18^, ^19^, ^20^ and identified it as an important factor associated with poor prognosis.^15^ Therefore, further studies are required in this regard.

Studying the association of other variables with HER2/neu overexpression in our study showed that the frequency of different scores did not differ based on patients' sex, age, smoking status and family history of cancer. The results of previous studies on the factors associated with HER2/neu overexpression are also controversial. Arcila et al reported more frequent HER2 mutations in never‐smokers but no associations with patients' sex, race or tumor stage.^17^ Mazieres et al have observed HER2/neu overexpression more frequently in women and non‐smokers.^12^ Furthermore, all of the eight cases with HER2/neu overexpression in the study by Li et al were never‐smokers, and 7/8 were women.^11^ Also, of 152 patients of never‐smoker adenocarcinomas, 12 had HER2/neu overexpression, 11 of whom were women.^21^ These findings are contrary to that of the present study, which could be because of the low frequency of female participants (16%) and non‐smokers (16%) in the present study. Another source of difference could be the tumor type, as all the above‐mentioned studies have only considered patients with adenocarcinoma. Generally speaking, most of the studies in this regard have focused on adenocarcinoma, and studies on other types of lung cancer are very few.^15^ Notably, we included patients with all tumor types to evaluate the significance of HER2/neu expression in different tumor types, but the number of samples in each subtype was few. As it has been demonstrated that the tumour's behaviour and characteristics vary significantly based on the tumor type, and the HER2/neu overexpression and its associated factors have to be more extensively studies in different tumor types.

Evidence suggests that the main mechanism of HER2 for induction of cell growth, proliferation, and survival in cancers is through phosphatidylinositol‐4,5‐bisphosphate 3‐kinase (PI3K) axis and mitogen‐activated protein kinase (MAPK) cascade, activated by the HER receptor.^8^ A recent study of NSCLC has demonstrated that cell proliferation and invasion induced by HER2 is related to the positive correlation of HER2 expression with the COX‐2 overexpression as well as MEK/ERK phosphorylation (via AKT signalling pathway).^22^ Others have also described how targeting HER2 can help the treatment of NSCLC.^23^, ^24^, ^25^ Meanwhile, as SCLC is considered the most common type with a rapid growth rate and poorer prognosis, more studies are required in this regard for this type of lung cancer.

One of the limitations of the present study was the non‐randomized inclusion of patients into the study, as well as the small number of samples in the subgroups, which increases the risk of bias and the influence of confounders on the results. Another important limitation of this study was the cross‐sectional nature of the study, which disabled us from studying the causal relationship between the variables or study the patients' prognosis.

## 5 CONCLUSION

The results of the present study emphasized the overexpression of HER2/neu in different types of lung cancer, which can be used further for therapeutic purposes. The results showed that HER2/neu was overexpressed not only in adenocarcinoma but also in other types, like SCC. Therefore, all subtypes of NSCLC should be considered for anti‐HER2 therapies, and further research is required for SCLC.

## CONFLICT OF INTEREST

The authors of the present study declare that have no conflict of interest.

## ETHICS STATEMENT

The protocol of the present cross‐sectional study was approved by the Ethics Committee of Kerman University of Medical Sciences (code: IR.KMU.RCE.1395.137). The ethical considerations of the latest version of Helsinki's Declaration on human studies were met throughout the study phases.

## AUTHOR CONTRIBUTIONS

Mohammadreza Lashkarizadeh manufactured the samples. Mahdiyeh Lashkarizadeh designed and performed the experiments and drafted the manuscript. Meead Nikian contributed to the design and implementation of the research, to the analysis of the results and to the writing of the manuscript. Maryam Kouhestani Parizi contributed to the revising the final manuscript. All authors discussed the results and contributed to the final manuscript.

## Data Availability

The data that support the findings of this study are available from the corresponding author upon reasonable request.
